# Genomic convergence between *Akkermansia muciniphila* in different mammalian hosts

**DOI:** 10.1186/s12866-021-02360-6

**Published:** 2021-10-29

**Authors:** Sharon Y. Geerlings, Janneke P. Ouwerkerk, Jasper J. Koehorst, Jarmo Ritari, Steven Aalvink, Bärbel Stecher, Peter J. Schaap, Lars Paulin, Willem M. de Vos, Clara Belzer

**Affiliations:** 1 Laboratory of Microbiology, Wageningen University, Wageningen, The Netherlands; 2 Laboratory of Systems and Synthetic Biology, Wageningen University, Wageningen, The Netherlands; 3 Finnish Red Cross Blood Service, Helsinki, Finland; 4 Max von Pettenkofer Institute of Hygiene and Medical Microbiology, Faculty of Medicine, LMU Munich, Munich, Germany; 5 Institute of Biotechnology, University of Helsinki, Helsinki, Finland; 6 Human Microbiome Research Program, Faculty of Medicine, University of Helsinki, Helsinki, Finland

**Keywords:** Comparative genomics, Phylogenetic analysis, Gut bacteria, Beneficial microbe, Gastrointestinal-tract

## Abstract

**Background:**

*Akkermansia muciniphila* is a member of the human gut microbiota where it resides in the mucus layer and uses mucin as the sole carbon, nitrogen and energy source. *A. muciniphila* is the only representative of the Verrucomicrobia phylum in the human gut. However, *A. muciniphila* 16S rRNA gene sequences have also been found in the intestines of many vertebrates.

**Results:**

We detected *A. muciniphila*-like bacteria in the intestines of animals belonging to 15 out of 16 mammalian orders. In addition, other species belonging to the Verrucomicrobia phylum were detected in fecal samples. We isolated 10 new *A. muciniphila* strains from the feces of chimpanzee, siamang, mouse, pig, reindeer, horse and elephant. The physiology and genome of these strains were highly similar in comparison to the type strain *A. muciniphila* Muc^T^. Overall, the genomes of the new strains showed high average nucleotide identity (93.9 to 99.7%). In these genomes, we detected considerable conservation of at least 75 of the 78 mucin degradation genes that were previously detected in the genome of the type strain Muc^T^.

**Conclusions:**

The low genomic divergence observed in the new strains may indicate that *A. muciniphila* favors mucosal colonization independent of the differences in hosts. In addition, the conserved mucus degradation capability points towards a similar beneficial role of the new strains in regulating host metabolic health.

**Supplementary Information:**

The online version contains supplementary material available at 10.1186/s12866-021-02360-6.

## Introduction

The gastrointestinal (GI) tract of vertebrates is colonized with a dense and diverse microbiota [1]. Several factors affect the gut microbiota composition of vertebrates including diet, host phylogeny and gut morphology [2]. The microbiota has had a large influence on animal evolution, and can be seen as an obligate and beneficial symbiont [3]. The main phyla representing the gut microbiota in mammals are Firmicutes, Bacteroidetes, Proteobacteria, Actinobacteria, and Verrucomicrobia [2]. The gut microbiota produces short chain fatty acids from degradation of otherwise indigestible components providing the host with the ability to digest a wider variety of available foods [2, 4]. The gut microbiota also produces vitamins and other beneficial substances that the host cannot synthesize [5].

Some microbiota members can flourish within the mucus layer, a glycan-rich and anaerobic environment offered by its host [6], including mucus-degrading specialist *Akkermansia muciniphila* [7]. *A. muciniphila* is the only representative of the Verrucomicrobia phylum in the human gut. Mucin utilization by *A. muciniphila* has been shown i) in vitro, where it grows on mucin as sole carbon and nitrogen source [7], ii) in vivo, where it scavenges mucin efficiently [8] and iii) in silico using a genome-scale model and omics analysis [9]. Recent mouse and human studies have demonstrated that intake of *A. muciniphila* has a series of health benefits, including improved barrier function, increased insulin sensitivity and reduction of obesity [10, 11]. In addition, mouse experiments have shown that while *A. muciniphila* can degrade mucin, its presence increases mucus production, mucus layer thickness and tight junction protein production [10, 12, 13].

*A. muciniphila* is abundantly present in the human intestinal tract, varying from 1 to 4% of the bacterial population in the colon [14]. Its abundance was found to be linked to a healthy status in humans [15]. Interestingly, *A. muciniphila* has also been detected in other mammals, such as the brown bear [16]. Furthermore, *A. muciniphila* has been detected in several small animals, such as the ground squirrel and Syrian hamster [17, 18]. In addition*, Akkermansia* spp. are widely spread in the GI tract throughout the animal kingdom, including mammals [2] and other vertebrates such as python [19, 20], zebra fish [21], chicken [21], and salmon [22]. Next to bacteria belonging to the genus *Akkermansia*, other Verrucomicrobia are detected in the animal GI tract of invertebrates including termites [23], ants [24], earthworm [25] and nematodes [26]. A few examples of Verrucomicrobia isolates from the gut environment are *A. muciniphila* [7], python isolate *A. glycaniphila* [20], and termite GI isolate *Diplosphaera colotermitum* [27].

*A. muciniphila* is able to colonize a broad range of hosts, despite differences in GI tract anatomy (simple, foregut, hindgut), diet (carnivore, omnivore, herbivore), host physiology and body temperature. This distribution might be an indication of co-evolution of this organism with its host. Therefore, we explored the presence and genomic divergence of Verrucomicrobia and *Akkermansia* spp. within different mammalian hosts.

## Results

### *A. muciniphila* and other Verrucomicrobia within the GI tract of different mammals

**Fig. 1:**
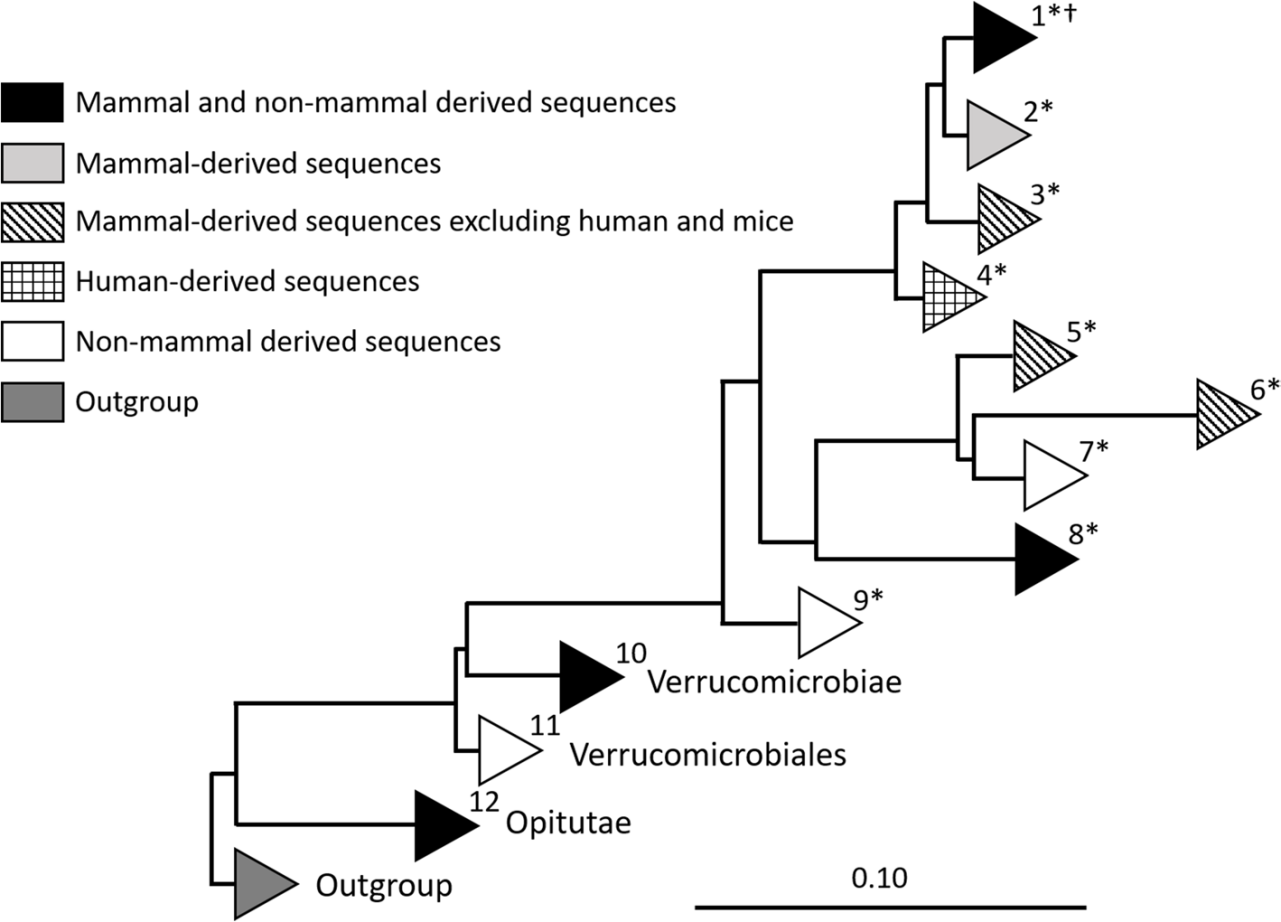
Verrucomicrobia diversity within samples from the GI tract. Schematic representation of all clades within the Verrucomicrobia phylum that contain intestinal obtained sequences based on the phylogenetic tree. (*) Clades containing *Akkermansia* sequences. (†) All newly isolated *A. muciniphila* strains are positioned in clade 1

Detailed analysis of Verrucomicrobia 16S rRNA gene sequences derived from SILVA database 138 [28] (> 1100 bp, pintail > 75) revealed that *A. muciniphila* is not the sole representative species of the Verrucomicrobia phylum in the GI tract of mammals. The phylogenetic tree was constructed using both the neighbor joining method and RAxML. Both trees showed similar output and the Verrucomicrobia-derived sequences could be grouped into 12 clades of which 9 clades contained samples obtained from the mammalian intestine (Fig. 1 and Fig. S1).

The first clade contains 1352 *A. muciniphila* like 16S rRNA sequences, not solely mammalian. In this clade, eight mammalian orders were detected: Proboscidea (African elephant), primates (human, gorilla, lemur, chimpanzee, pygmy loris), Carnivora (cheetah), Sirenia (Dugong), Cingulata (armadillo), Rodentia (rat, mice and thirteen-lined ground squirrel), Artiodactyla (eland, pig, cow (rumen fluid)), Perissodactyla (horse). The non-mammalian sequence present in this clade was derived from a chicken. In addition, the 16S rRNA sequences of *A. muciniphila* strains isolated in this study were positioned in clade 1 (Fig. 1).

Five clades solely contained mammalian sequences, including clade 2, 3, 4, 5 and 6. Clade two contains 277 sequences, mainly derived from primates (human) and Rodentia (mice) and one sequence derived from Sirenia (dugong). Interestingly, sequences derived from the snub-nosed monkey formed a separate clade (3) within the phylogenetic tree, as well as 57 human-derived sequences in clade 4. Other clades solely consisting of mammalian derived sequences were clade 5 and 6. Clade 5 contained 201 sequences derived from 6 mammalian orders, including Proboscidea (African elephant), Artiodactyla (cow, okapi, buffalo, babirusa, warty pig, gazelle, takin, giraffe, przewalskii gazelle, springbok), Diprotodontia (kangaroo), Rodentia (capybara, Prevost’s squirrel), Perissodactyla (horse, wild ass, rhinoceros, zebra), Chiroptera (flying fox). Clade 6 contained 20 sequences from mammalian orders Lagomorpha (rabbit) and Proboscidea (elephant). Sequences derived from animals both living in captivity and in the wild were represented in all animal-containing clades excluding mice. To be able to compare the similarity (%) of type strain *A. muciniphila* MucT to the different clades, *A. muciniphila* MucT was compared to representative sequences of each clade. The similarities and amount of representatives per clade are shown in Table 1.

**Table 1:** GI tract obtained Verrucomicrobia sequences of clades 1–12 corresponding to Fig. 1

Clade	Total amount of sequences in clade	Taxonomy	Host	Similarity (%) to MucT lower limit	Similarity (%) to MucT upper limit	Amount representative sequences
1	1352	Genus: Akkermansia	Human (786), other primates (89), Proboscidea (17), Carnivora (4), Sirenia (2), Cingulata (2), rodentia (440), Artiodactyla (6), Perissodactyla (3) and Galliformes (3)	91.91	100	15
2	277	Genus: Akkermansia	Human (111), Rodentia (165) and Sirenia (1).	95.66	99.09	10
3	4	Genus: Akkermansia	Primates (4)	98.30	98.84	4
4	57	Genus: Akkermansia	Human (57)	94.18	98.41	5
5	201	Genus: Akkermansia	Proboscidea (1), Artiodactyla (142), Diprotodontia (30), Rodentia (3), Perissodactyla (24) and Chiroptera (1)	85.15	90.69	17
6	20	Genus: Akkermansia	Rodentia (19) and Proboscidea (1)	86.25	89.22	4
7	12	Genus: Akkermansia	Fish gut sequences (12)	89.41	90.22	3
8	5	Genus: Akkermansia	Squamata (4) and Sirenia (1)	94.03	94.11	2
9	7	Genus: Akkermansia	Invertebrates (7)	92.74	93.06	2
10	45	Order: Chtoniobacterales, Methylacidiphales and Verrucomicrobiales	Human (UC patients) (2), moth larvae (1), earthworm (37), termite (1), grass carp (2) and ascidian (2).	82.12	86.52	7
11	63	Order: Verrucomicrobiales	earthworm (24), ascidian sea squirt (29), sea cucumber (1), sea horse (1), olive flounder (1), small abalone (2), brown surgeonfish (1), black surgeonfish (1) and grass carp (1), squat lobster (2)	83.37	87.20	8
12	89	Order: Opitutales	Termites (11), ants (6), black millipede (1), cockroaches (2), ascidian (43), olive flounder (1), royal panaque (1), flying fox (2), baboon (2), eastern black and white colobus (12), Sumatran orang-utan (3), red kangaroo (3), capybara (1) and European rabbit (1).	77.95	82.35	10

Other mammalian GI tract derived sequences that belonged to the Verrucomicrobia phylum were found within clade 8, 10 and 12. Clade 8 consisted of only five sequences in total, four derived from a python (*A. glycaniphila*) and one sequence from the mammalian order Sirenia (Dugong). Clade 10 contained two sequences that belong to the Prosthecobacter genus obtained from intestinal samples of UC patients (Fig. 1 and Table 1). Furthermore clade 12 contained sequences from mammalian orders Chiroptera (flying fox) and primates (hamadryas baboon and eastern black and white colobus and Sumatran orangutan), Diprotodontia (red kangaroo), Rodentia (capybara) and Lagomorpha (European rabbit) belonging to the class Opitutae. The remaining Verrucomicrobia clades (7, 9 and 11) solely contained 16S rRNA gene sequences derived from the non-mammal Animalia GI tract (Fig. 1 and Table 1).

### Verrucomicrobia prevalence in fecal samples of different mammals

**Fig. 2:**
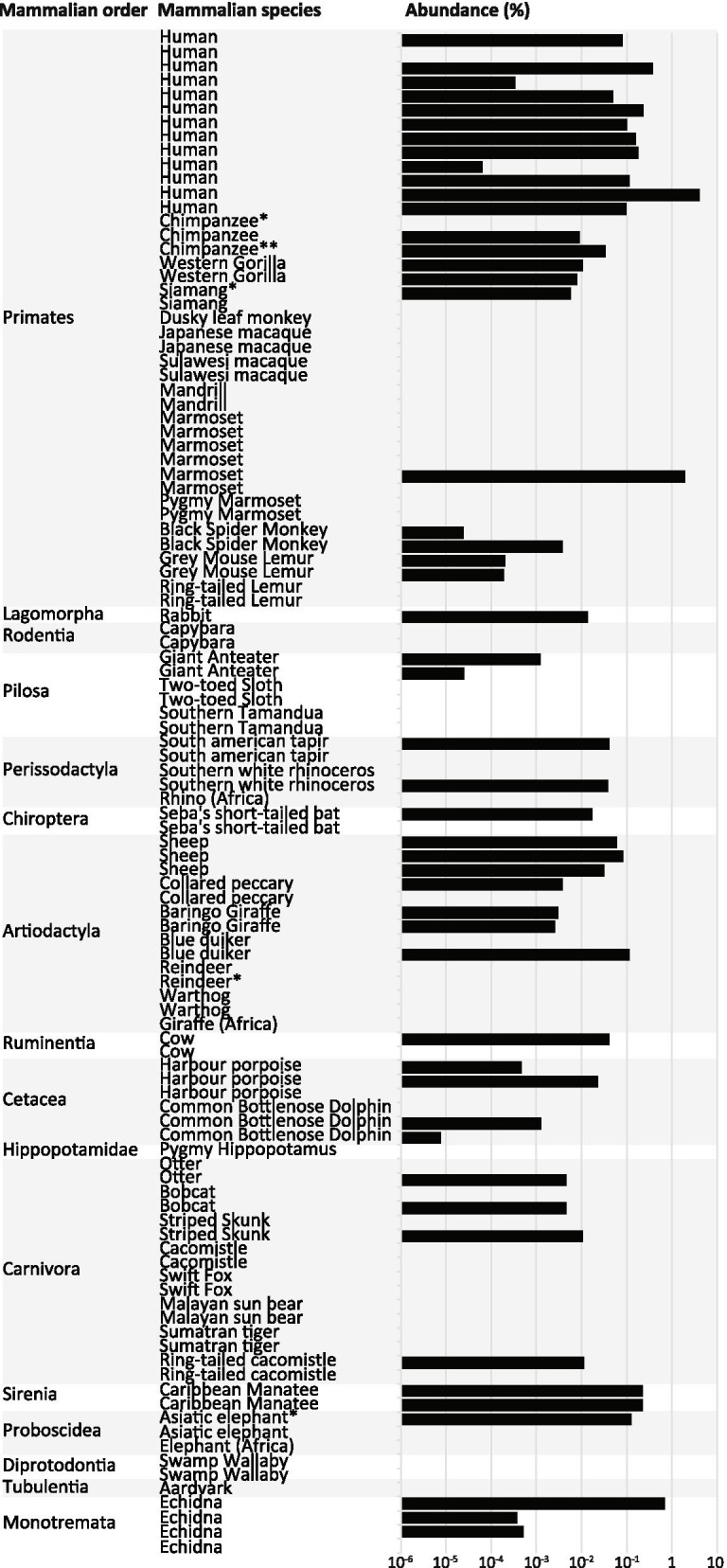
Prevalence, abundance and phylogeny of *A. muciniphila*. Orders are depicted on the vertical axis following the phylogeny of mammals (for primates [29]). (*) Samples from which pure isolates were obtained. Abundance of the *Akkermansia* genus was determined using qPCR

Fecal samples of 108 different animals belonging to 47 species of 16 mammalian orders were collected. The prevalence of *A. muciniphila* was determined by quantitative PCR (qPCR) on all samples. Amplicons were generated with *A. muciniphila*-specific primers in 50 out of 108 samples, with abundances up to 4% (Fig. 2). In addition, 16S rRNA gene sequencing of Verrucomicrobia resulted in Verrucomicrobia sequences derived from the Caribbean manatee, echidna, Western gorilla and otter. These sequences were added to the phylogenetic tree shown in Fig. 1, in which they were positioned in clade 1 (Caribbean manatee), clade 5 (Western gorilla and echidna) and clade 10 (otter).

### New *A. muciniphila* isolates show low physiologic divergence

**Fig. 3:**
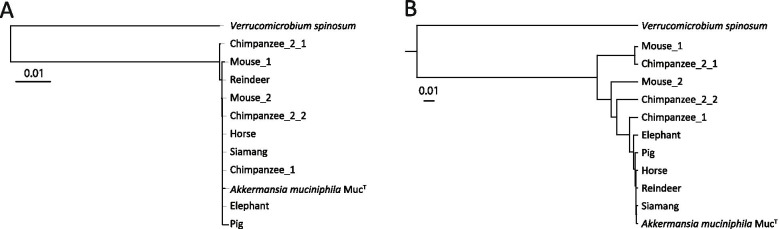
Phylogeny of the new isolates. **A** Phylogeny of the new isolates based on 16S rRNA gene sequence, aligned in ARB using NJ. Bar represents 1% sequence divergence. **B** Phylogeny of the new isolates based on the presence of domains in the draft genomes. Bar represents 1% sequence divergence. *A. muciniphila* MucT, and *V. spinosum* DSM 4136 are used as reference

**Fig. 4:**
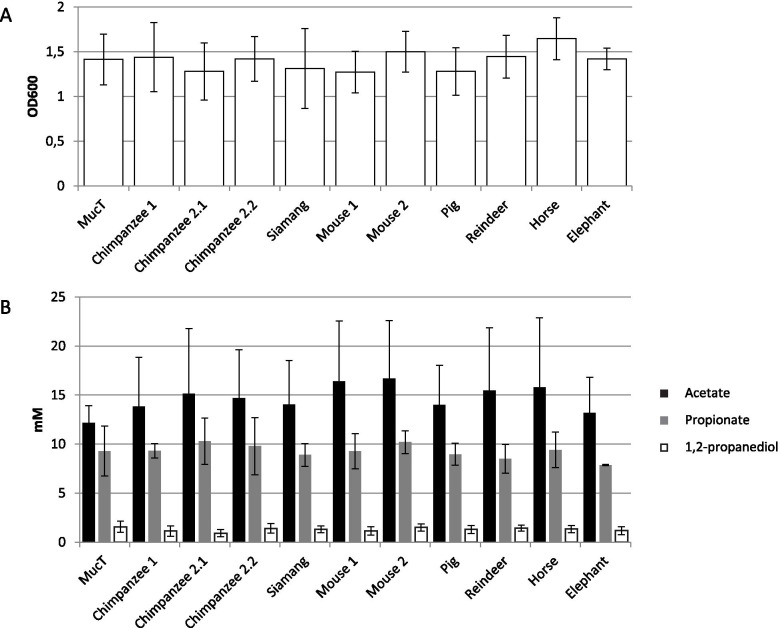
Physiology on mucin-based medium. **A** Maximum OD600 reached when grown on a mucin-based medium. **B** The SCFA profile when grown on a mucin based medium

Ten new *A. muciniphila* isolates were obtained from fecal samples of the chimpanzee, siamang, mouse, pig, reindeer, horse, and elephant. The 16S rRNA gene sequence of these ten isolates was determined, and showed high similarity (> 99.9%) to the 16S rRNA gene sequence of *A. muciniphila* MucT (Fig. 3A). All new *A. muciniphila* strains had small and oval shaped cells of approximately 700 nm in length, as described previously for the type strain [7]. All cells stained Gram-negative, grew in single cells, doublets, and aggregates in mucin medium. The cell growth (determined by OD600) and short chain fatty acid (SCFA) production (determined by high performance liquid chromatography (HPLC)) in a mucin-based medium was similar to the type strain *A. muciniphila* MucT (Fig. 4). In addition, the strains had similar growth rates, and produced similar amounts of acetate, propionate and 1,2-propanediol. Taking into consideration the morphologic, physiologic and 16S rRNA gene sequence similarity, all strains should belong to *A. muciniphila* species.

### Low genomic divergence between 10 new *A. muciniphila* isolates

The genomic DNA of all newly obtained isolates were sequenced and assembled into draft genomes (Table 2) consisting of 25–215 contigs. Isolates had genome sizes in the range of *A. muciniphila* MucT (2.7 Mb), although the genomes of Chimpanzee 2_1, Chimpanzee 2_2, and Mouse 1 were slightly larger (2.9 Mb) (Table 2). This was also reflected in the total predicted gene count (Table 2). All isolates had comparable GC content ranging from 55.2 to 55.9 (Table 2). The average nucleotide identity (ANI) was > 99.7% for 7 isolates (from chimpanzee 1, siamang, mouse 1, pig, reindeer, horse, and elephant). The ANI was lower for the isolates from chimpanzee 2_1 (93.9%), chimpanzee 2_2 (97.4%), and mouse 2 (93.9%). The BLAST similarity and the number of SNPs of these three genomes were also in line with these results. This indicates that these three isolates are phylogenetically more distant. The ANI BLAST between all new isolates is shown in Table S1. The phylogeny based on the domain presence in the genomes was constructed and reflected the 16S rRNA gene phylogeny (Fig. 3B and Table S2). In addition the pan-genome has been determined as shown in Fig. S2.

**Table 2:** General genome characteristics

Strain	Coverage	Contigs	Genome size (Mbp)	GC content (%)	Total gene count	Comparison with ***A. muciniphila*** Muc^**T**^		
						ANI (%)	BLAST similarity (> 5 kb) (%)	SNP
**Chimpanzee 1**	222	77	2.6	55.7	2233	99.98	99.99	0
**Chimpanzee 2_1**	150	185	2.9	55.9	2629	93.87	94.39	82
**Chimpanzee 2_2**	208	120	2.9	55.2	2524	97.38	97.83	124,644
**Siamang**	212	110	2.7	55.8	2291	99.94	99.99	53,418
**Mouse 1**	179	215	2.9	55.8	2585	93.85	93.49	128,793
**Mouse 2**	120	25	2.7	55.5	2330	99.85	98.91	25,020
**Pig**	185	70	2.8	55.8	2384	99.98	99.99	8
**Reindeer**	127	104	2.7	55.7	2303	99.94	99.99	14
**Horse**	271	90	2.8	55.7	2369	99.95	99.99	13
**Elephant**	193	188	2.7	55.7	2361	99.99	99.99	15

**Fig. 5:**
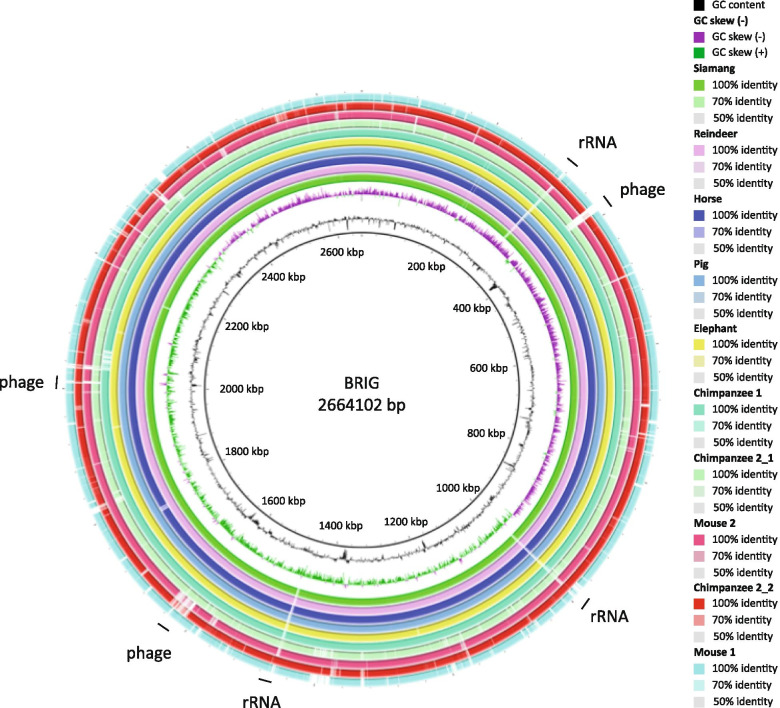
BRIG genome comparison of draft genomes of new isolates to *A. muciniphila* MucT. Genome comparison to the type strain *A. muciniphila* MucT as reference based on sequence similarity. From inside to outside: the first ring describes the GC content, the second ring describes the GC skew, the next ten rings describe the similarity of the represented genomes to *A. muciniphila* MucT as explained in the legend. On the outside both the rRNA operons and the phage remnants are indicated

Comparing all draft genomes of the new isolates to the complete genome of *A. muciniphila* MucT using BLAST Ring Image Generator (BRIG) showed the low genomic divergence (Fig. 5). Several potential phage remnants were identified that showed different GC content or GC skew and were not conserved among all isolates. Moreover, on 3 points in the draft genome there was a gap, likely because of the presence of one of the 3 rRNA operons that interfered with the draft genome sequence assembly.

Mucin degradation and utilization proteins of all isolates were individually analyzed for sequence divergence. In addition to the 61 mucin-degrading proteins predicted to be excreted [30], 18 other genes were predicted to be involved in mucin degradation and utilization. The seven isolates (from chimpanzee 1, siamang, mouse 1, pig, reindeer, horse, and elephant) that had the highest sequence similarity compared to strain MucT harbored the same 78 mucin degradation and utilization proteins (Additional Table S3), though the draft genome isolate obtained from a horse lacked one α-L-fucosidase (Amuc_1699). In comparison with the complete genome of the type strain MucT, we found that the draft genome of chimpanzee strain 2_2 lacked genes for two glycosyl hydrolases (Amuc_1637 and Amuc_1120) and the PfkB (Amuc_0075), which is annotated to be involved in fructose degradation. In comparison with the complete genome of the type strain MucT, we found that the draft genome of chimpanzee strain 2_1 lacked genes for two glycosyl hydrolases (Amuc_1120 and Amuc_0824) and one α-N-acetylglucosaminidase (Amuc_1220) as compared to the type strain. In comparison with the complete genome of the type strain MucT, we found that the draft genome of mouse strain 2 solely lacked the gene for one glycosyl hydrolase (Amuc_1637).

## Discussion

*Akkermansia muciniphila* is an abundant member of the healthy human intestine, and colonizes the mucus layer that lines the intestinal epithelial cells. Apart from human, *Akkermansia* 16S rRNA gene sequences can be detected in intestinal samples of many vertebrates. *A. muciniphila* is the only cultured representative of the Verrucomicrobia phylum obtained from the human GI tract and was isolated from a fecal sample of a healthy adult. We determined the prevalence of Verrucomicrobia using the SILVA database, the clone libraries of Verrucomicrobia specific amplicons, and performed qPCR on 108 fecal samples collected for this study. In addition, we obtained ten new *A. muciniphila* isolates from non-human mammals that were characterized by determining their draft genome.

Our results indicate that *A. muciniphila* is not the sole species belonging to the phylum Verrucomicrobia that colonizes the GI tract of mammals. Clade 5 and 6, that contained sequences derived from fecal samples of a wide variety of mammals excluding human and mice, were found to be closely related to *A. muciniphila* MucT. The 16S rRNA gene sequence similarities compared to MucT ranged between 85.15–90.69% and 86.25–89.22% for clade 5 and 6, respectively. In addition, more distantly related Verrucomicrobia sequences were detected in fecal samples. Verrucomicrobia sequences belonging to the Prosthecobacter family (85% similarity) were detected in GI samples of UC patients. Furthermore, Verrucomicrobia sequences belonging to the Opitutae class have been detected in the following mammals: flying fox, European rabbit, capybara, red kangaroo, Sumatran orangutan, hamadryas baboon and, eastern black and white colobus.

Clade 5 and 6 contain only non-human mammalian sequences. Our isolation effort did not result in any of these *Akkermansia* species. This could mean that mucin is not the main nutritional source for these organisms or that there are differences in host mucin and therefore in mucus degradation by *Akkermansia*. However, more research is needed to investigate the type of mucins produced and secreted by the gastrointestinal tract of different mammals to confirm this hypothesis. Recently, co-colonization with multiple *A. muciniphila* strains has been described to be possible inhealthy human individuals [31]. It would be of interest to investigate whether *A. muciniphila* and a species from clade 5 or 6 could co-colonize in a mucin-dominated environment within the gut.

Our results confirm earlier reports that *A. muciniphila* is widely spread throughout mammals [21]. Moreover, we provide a comparative genomic analysis of new *A. muciniphila* isolates. By combining 16S rRNA gene database sequences and quantitative PCR, we could detect *Akkermansia* in animals that belong to 15 out of 16 mammalian orders included in this study. The *Akkermansia* 16S rRNA gene sequences present within different animals were highly similar. This suggests that *Akkermansia* is highly conserved and only minor changes upon co-evolution with its different host species occurred.

The qPCR analysis did not confirm the presence of *A. muciniphila* in two of the samples from which new *A. muciniphila* isolates were obtained (chimpanzee 1 and reindeer). The Verrucomicrobia PCR did confirm the presence of Verrucomicrobia in these samples. Possible inhibition of qPCR amplification might be the reason that *A. muciniphila* was not detected in these samples.

Although for some orders only few animals were tested, there does not seem to be a correlation between the abundance of *Akkermansia spp.* and the host phylogeny. A possible explanation for this high degree of conservation could be horizontal spread of the microorganism among animals in captivity due to close contact with humans as most samples were taken in the Dutch zoos. However, we could confirm that *Akkermansia spp.* and other Verrucomicrobia can also be detected in fecal samples of animals that live in the wild, both from our and a previous study [2]. The spread of *Akkermansia spp.* is not restricted to any geographical location, since highly similar 16S rRNA gene sequences were found in fecal samples taken from different parts of the world, more specific in rural Africa [2], rural Australia (this study), North-America [2, 32, 33] Asia [34] and Europe (this study) [7]. Based on the observation that highly similar 16S rRNA gene sequences were found to be present within the different animals, we hypothesize that *Akkermansia spp.* are highly conserved within its different hosts. Potentially because only minor changes upon co-evolution with its different host species are needed to colonize the mucosal niche.

The presence of *A. muciniphila* in the mammalian GI-tract has also been assessed by reconstructing metagenome assembled genomes from datasets of human, mouse and pig gut microbiomes [35]. In line with our findings, the presence of *A. muciniphila* in the mammalian gut was found to be globally distributed. In this study, we used culturing techniques to isolate new *A. muciniphila* strains from the mammalian GI-tract. However, metagenome assembled genomes of *Akkermansia* spp. that could not be obtained by culturing techniques in this study, may give more insight into the function of these strains and their ability to degrade mucus in the mammalian gut.

Mucin proteins are conserved among mammals, and even within the chordate phylum. Mucin glycoproteins are rich in proline, threonine and serine and are highly glycosylated [36]. These properties enable *A. muciniphila* to use mucin as both nitrogen and carbon source [37]. It is not known how the mucin proteins are glycosylated in the numerous vertebrates, but regardless of the potential glycosylation patterns the genomes of the *A. muciniphila* isolates encode many enzymes that can cleave a wide variety of glycan chains. Differences in the presence of mucus degradation genes in comparison to the type strain were only detected in three out of ten isolates. The genes lacking in these genomes may not directly have an effect on the mucus degrading capability, since other glycosyl hydrolases, α-L-fucosidases and N-acetylglucosaminidases are also present in the genome of *A. muciniphila*. Furthermore, it is important to note that the genomes of the new isolates were not closed. Therefore, it is a possibility that these genes are present in the genome but not detected in our analysis. Overall, this data suggests that *A. muciniphila* is a mucin-degrading specialist that has the potential to colonize different mammals regardless of their potential differences in mucin structure.

We did not observe indications for animal-species specific colonization when connecting the sequences of the *Akkermansia* clades with the hosts of origin. This contrasts what has been described for *H. pylori*, a well-studied mucosal pathogen that is mainly found in human and in very narrow range of other hosts [38]. Testifying for the adaptation of *H. pylori* to the human host is the observation that its genome can be linked to human migration over our planet [39].

## Conclusions

Our findings indicate that *A. muciniphila* is frequently colonizing the GI tract of mammals. In this study, we isolated 10 new *A. muciniphila* strains from feces of chimpanzee, siamang, mouse, pig, reindeer, horse and elephant. All new *A. muciniphila* isolates grew on mucin as sole carbon and nitrogen source suggesting that representatives of this species colonize the mucus layer of its host and therefore seem not be affected by the host diet or physiology. The low genomic divergence observed in the new strains may indicate that *A. muciniphila* favors mucosal colonization independent of the differences in hosts. In addition, the conserved mucus degradation capability points towards a similar beneficial role of the new strains in regulating host metabolic health.

## Methods

### Sample collection

Fecal samples from animals were obtained at three Dutch zoo’s: Burgers Zoo (Arnhem, The Netherlands), Dolfinarium (Hardewijk, The Netherlands), Natura Artis Magistra (Amsterdam, The Netherlands), Plankendael (Antwerp, Belgium), animal facilities of Wageningen UR (Wageningen, The Netherlands), animal facilities of Erasmus MC (Rotterdam, The Netherlands), animal facility of the Institute of Microbiology, (ETH Zurich, Switzerland), Animal facilities of Leiden University Medical Centre (Leiden, The Netherlands), from pets living at Dutch homes, but also from wild animals that live in either rural Africa or rural Australia. Mouse 2 has been deposited under the name *A. muciniphila* YL44, DSM26127 (https://www.dsmz.de/catalogues/dzif-sammlung-der-dsmz/maus-mikrobiomliste.html). Additional information about the samples can be found in Table S4.

### Isolation and growth conditions

Approximately 0.2 g of fecal sample was taken and dissolved in anaerobic PBS (pH 7) containing 0.5 g/l of cysteine-HCL within 24 h of defecation. A fraction of all samples was used to prepare a glycerol (25%v/v) stock and stored at − 80 °C. The other fraction of all samples were 10-fold diluted in anaerobic mucin medium, composed of a bicarbonate-buffered basal medium [7] with a pH of 6.5–7.0, supplemented with 0.5% (vol/vol) purified and dialyzed hog gastric mucin (Type III, Sigma) as sole carbon and nitrogen source as described previously [40]. All incubations were performed until growth was observed at 37 °C in 30 ml serum bottles, containing 10 ml mucin media, sealed with butyl rubber stoppers under anaerobic conditions provided by a gas phase of 1.5 atm N2/CO2 (80:20 vol/vol). Enrichment was achieved by repeated serial dilutions. After this primary enrichment, the strains were purified by repeated plating of single colonies onto anaerobic mucin medium agar (0.8% (w/v) agar (Bacto Agar, BD), only selecting *Akkermansia-*like colonies based on previously described morphology [7] and of which an *Akkermansia-*specific PCR [14] was found positive. The Short-chain fatty acids in cultures containing the purified strains were measured using a Thermo Electron spectrasystem HPLC equipped with an Agilent Metacarb 67H column. Purified strains were stored in mucin medium containing glycerol (25% v/v) at − 80 °C. DNA was extracted using the Masterpure™ Gram Positive DNA Purification Kit (Epicentre®).

### *A. muciniphila* 16S rRNA gene abundance

Quantitative PCR amplification was performed as previously described [14] with minor modifications: samples were analyzed in a total volume of 10 μl consisting of 1 x iQ SYBR Green Supermix (BioRad), 200 nM forward primer AM1: CAGCACGTGAAGGTGGGGC [14] or 1369F: CGGTGAATACGTTCYCGG [41], 200 nM reverse primer AM2: CCTTGCGGTTGGCTTCAGAT [14] or 1492R: CGGCTACCTTGTTACGAC [42], 1 x VisiBlue Master Mix colorant (Tataabiocenter), and 0.2 ng/μl sample DNA, Nuclease-Free Water (Promega) was added to 10 μl. The primerset including AM1 and AM2 specifically amplifies *A. muciniphila* DNA and the primerset including 1369F and 1492R is a general 16S rRNA primerset to determine the total abundance. All reactions were performed in triplicates in a BioRad CFX-384 device (Veenendaal, The Netherlands). Standard curves of 16S rRNA from *A. muciniphila* cloned into pGMTeasy vector (Promega) were prepared, corresponding to a range from 10^8^ to 10^0^ cells. The quality of the standard curves were assessed using qPCR. The abundance of *A. muciniphila* 16S rRNA genes was determined by dividing the amount of *Akkermansia* 16S rRNA gene amplicon by that obtained from total 16S after correcting for the 16S rRNA gene copy of *A. muciniphila* (3 copies), and the average number of 16S rRNA genes (4.1 copies) in intestinal bacteria [43]. The starting quantity (SQ) values used for the calculations are available in Table S5.

### 16S rRNA gene sequencing of Verrucomicrobia

DNA obtained from the fecal samples was amplified in a final volume of 25 μl consisting of 1 x Green GoTaq reaction buffer (Promega), 200 nM of each dNTPs (Promega), 200 nM forward primer VER_37: TGGCGGCGTGGWTAAGA [44], 200 nM of reverse primer VER_673: TGCTACACCGWGAATTC [44], 1 U GoTaq DNA polymerase (Promega), Nuclease-Free Water (Promega) was added to obtain a total volume of 25 μl. Samples were amplified with a Dinxperlo BV G Storm thermocycler (Somerton Biotechnology) with the following program: Denaturation at 95 °C for 5 min, followed by 35 cycles of denaturation at 95 °C for 30 s, annealing at 50 °C for 30 s, extension at 72 °C for 1 min, and a final extension step at 72 °C for 10 min. The amplicons were purified using a High pure PCR Cleanup micro kit following the manufacturer’s protocol (Roche, Woerden, the Netherlands). Ligation of these amplicons in pGEMTeasy vector system as described by the manufacturer (Promega) and subsequent transformation into *E. coli* XL1-blue competent cells (Agilent Technologies). Inserts were sequenced at GATC (Biotech, Konstanz, Germany) using the flanking binding sites for T7: TATTTAGGTGACACTATAG and SP6: TAATACGACTCACTATAGGG. Vector, primers and low quality ends of the sequences were trimmed using DNA-baser v.354. 16S rRNA gene sequences were aligned using the SINA online alignment services [45] and subsequently imported into ARB [46].

### 16S rRNA gene database mining and phylogenetic tree construction

All intestinal Verrucomicrobia sequences > 1100 bp, with pintails > 75 were downloaded from the SILVA database version 138. The isolation source and host organism, if lacking, were retrieved from the original publications if possible and added to the designated fields in the database. All analysis concerning 16S rRNA gene sequences used for data mining performed on this dataset. The selected outgroup for the phylogenetic analysis consisted of 13 sequences from three phyla: Lentisphaerae, Omnitrophica and Chlamydiae. All 16S rRNA based phylogenetic analysis were performed with a single trimmed alignment file. The phylogenetic tree was constructed in ARB (version 5.3-org-8209) using a randomized axelerated maximum likelihood (RAxML) method (version 7.0.3) and a 40% positional conservatory filter [46]. Depending on the amount of sequences in each clade, up until 20 representatives of each clade were selected for sequence similarity comparisons of each clade to *A. muciniphila* MucT.

### DNA isolation and genome sequencing

High molecular weight genomic DNA was extracted from overnight-grown cultures as previously described [47]. DNA quality and concentrations were determined by spectrophotometric analysis using NanoDrop equipment (Thermo Scientific) and by electrophoresis on a 1% agarose gel. DNA was stored at − 20 °C until subsequent sequencing.

Genome sequencing was carried out at the Institute of Biotechnology, University of Helsinki (Finland). A MiSeq library was generated and sequenced on an Illumina MiSeq Personal Sequencer with 250 bp paired-end reads and an insert size of 500 bp. Reads were assembled using Ray (k-mer 101) [48].

### Genome annotation

Annotation was carried out with an in-house pipeline consisting of Prodigal v2.5 for prediction of protein coding DNA sequences [49], InterProScan 5RC7 for protein annotation [50], tRNAscan-SE v1.3.1 for prediction of tRNAs [51] and RNAmmer v1.2 for prediction of rRNAs [52]. Additional protein function predictions were derived via BLAST identifications against the UniRef50 [53] and Swissprot (UniProt, 2014) databases (download August 2013). Subsequently, the annotation was further enhanced by adding EC numbers via PRIAM version 2013-03-06 [54]. Non-coding RNAs were identified using rfam_scan.pl v1.04, on release 11.0 of the RFAM database [55]. CRISPRs were annotated using CRISPR Recognition Tool v1.1 [56]. A further step of automatic curation was performed by weighing the annotation of the different associated domains, penalizing uninformative functions (e.g. “Domain of unknown function”), and prioritizing functions of interest (e.g. domains containing “virus”, “bacteriophage”, “integrase” for bacteriophage related elements; similar procedure for different other functions).

### Pan-genome analysis

To determine the pan-genome, the genomes were annotated using Prokka [57]. Subsequently, Roary was used to obtain the core-genome alignment [58]. Based on this information, a maximum likelihood phylogenetic tree was constructed. The phylogenetic tree and the core-genome alignment were combined in Phandango to visualize the results [59].

### 16S rRNA gene sequence retrieval

For each organism the 16S rRNA reads were retrieved by filtering the FASTQ file through sortmeRNA using default settings while only using the 16S SILVA 118 database [60]. The obtained reads where then assembled using IDBA_UD into a 16S rRNA gene contig and used for further analysis [61].

## Supplementary Information

**Additional file 1:** **Figure S1.** Original detailed randomized axelerated maximum likelihood (RAxML) tree.

**Additional file 2:** **Table S1.** ANI BLAST and aligned percentage between all new *A. muciniphila* isolates.

**Additional file 3:** **Table S2.** Overview of the functional domains of the type strain and all new *A. muciniphila* isolates.

**Additional file 4:** **Figure S2.** Pan-genome of the type strain and all new *A. muciniphila* isolates visualized using Phandango.

**Additional file 5:** **Table S3.** Mucin degradation and utilization genes. Depicted are the sequence similarities of the genes from the newly obtained strains compared to the genes of strain Muc^T^.

**Additional file 6:** **Table S4.** Metadata of obtained fecal samples and isolates.

**Additional file 7:** **Table S5.** Starting quantity (SQ) values of both *Akkermansia* and the general 16S rRNA qPCR runs.

## Data Availability

The whole genome sequencing data of the *A. muciniphila* isolates obtained during the current study have been uploaded to the BioProject PRJEB21068 repository.

## Abbreviations

GI tract: Gastrointestinal tract
OTUs: Operational Taxonomic Units
SCFA: Short Chain Fatty Acids
HPLC: high performance liquid chromatography
ANI: Average Nucleotide Identity
BRIG: BLAST Ring Image Generator
RAxML: Randomized Axelerated maximum likelihood
