# Supplementary material for: Genomic convergence between Akkermansia muciniphila in different mammalian hosts
Source: BMC Microbiol. 2021 Oct 29;21:298. doi: 10.1186/s12866-021-02360-6 (PMC8555344; doi:10.1186/s12866-021-02360-6)
Supplement: Supplementary file 4 — Additional file 4: Figure S2. Pan-genome of the type strain and all new A. muciniphila isolates visualized using Phandango. [file 12866_2021_2360_MOESM4_ESM.pdf]

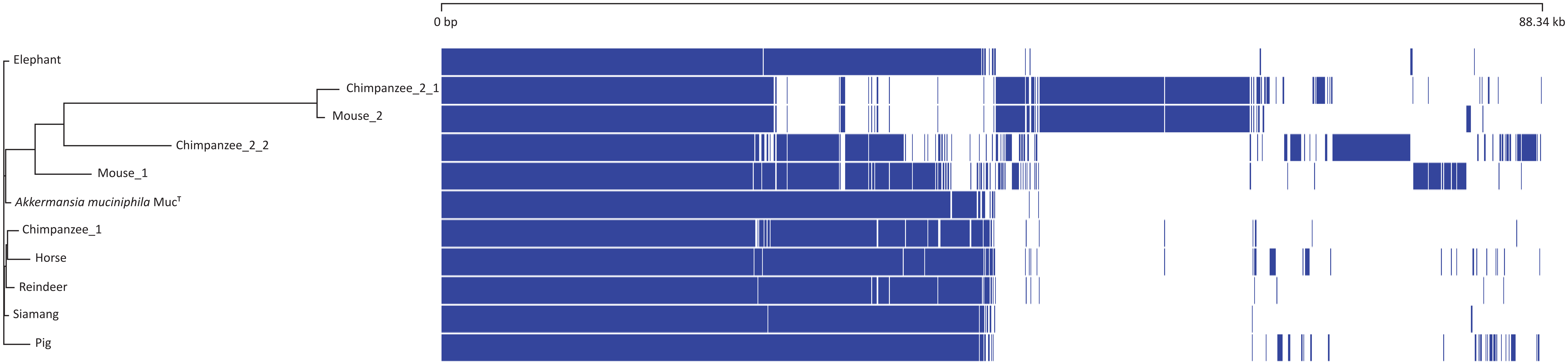

Figure S2: Pan genome of the type strain and all new *A. muciniphila* isolates visualized using Phandango
