# Supplementary material for: Genomic convergence between Akkermansia muciniphila in different mammalian hosts
Source: BMC Microbiol. 2021 Oct 29;21:298. doi: 10.1186/s12866-021-02360-6 (PMC8555344; doi:10.1186/s12866-021-02360-6)
Supplement: Supplementary file 1 — Additional file 1: Figure S1. Original detailed randomized axelerated maximum likelihood (RAxML) tree. [file 12866_2021_2360_MOESM1_ESM.pdf]

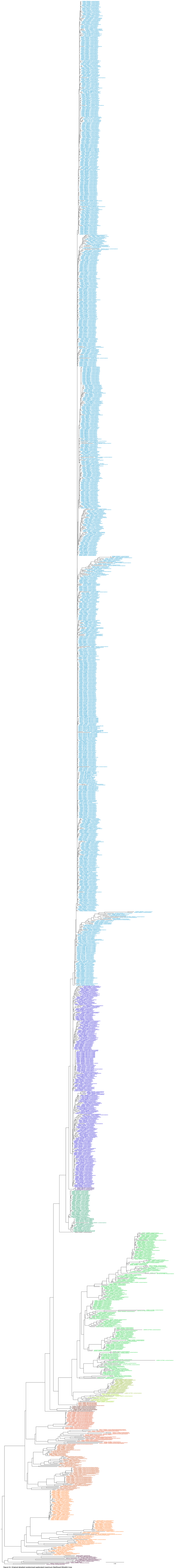

Figure S1: Original detailed randomized axelerated maximum likelihood (RAxML) tree
